# From Malignant Progression to Therapeutic Targeting: Current Insights of Mesothelin in Pancreatic Ductal Adenocarcinoma

**DOI:** 10.3390/ijms21114067

**Published:** 2020-06-06

**Authors:** Christopher Montemagno, Shamir Cassim, Jacques Pouyssegur, Alexis Broisat, Gilles Pagès

**Affiliations:** 1 Département de Biologie Médicale, Centre Scientifique de Monaco, 98000 Monaco, Monaco; shamir_cassim@yahoo.fr (S.C.); jacques.pouyssegur@unice.fr (J.P.); gilles.pages@unice.fr (G.P.); 2 Institute for Research on Cancer and Aging of Nice, Université Cote d’Azur, CNRS UMR 7284, INSERM U1081, Centre Antoine Lacassagne, 06200 Nice, France; 3 Laboratoire Radiopharmaceutiques Biocliniques, INSERM, 1039-Université de Grenoble, 38700 La Tronche, France; alexis.broisat@inserm.fr

**Keywords:** PDAC, mesothelin, CAR T-cells, antibody-based therapy, vaccines

## Abstract

Pancreatic ductal adenocarcinoma (PDAC), accounting for 90% of all pancreatic tumors, is a highly devastating disease with poor prognosis and rising incidence. The lack of available specific diagnostics tests and the limited treatment opportunities contribute to this pejorative issue. Over the last 10 years, a growing interest pointing towards mesothelin (MSLN) as a promising PDAC-associated antigen has emerged. The limited expression of MSLN in normal tissues (peritoneum, pleura and pericardium) and its overexpression in 80 to 90% of PDAC make it an attractive candidate for therapeutic management of PDAC patients. Moreover, its role in malignant progression related to its involvement in tumor cell proliferation and resistance to chemotherapy has highlighted the relevance of its targeting. Hence, several clinical trials are investigating anti-MSLN efficacy in PDAC. In this review, we provide a general overview of the different roles sustained by MSLN during PDAC progression. Finally, we also summarize the different MSLN-targeted therapies that are currently tested in the clinic.

## 1. Introduction

Pancreatic ductal adenocarcinoma (PDAC) is the most prevalent neoplastic disease of the pancreas representing 90% of pancreatic malignancies [1]. PDAC is one of the most aggressive malignancies, representing the fourth leading cause of cancer-related deaths in men and women worldwide in 2018 [2,3]. Its incidence is rising, and PDAC is projected to surpass breast, colorectal and prostate cancers, and to become the second deadliest malignancy by the year 2030 [4,5]. Indeed, projections indicate that a more than two-fold augmentation of PDAC cases is expected in the next ten years. One reason for this dramatic increase is its relationship with obesity and type-2 diabetes, two modern health drivers in PDAC etiology [6,7]. Moreover, alcohol and tobacco habits also increase the risk of PDAC development [8,9]. Finally, germline mutations in the *BRCA1, BRCA2*, *TP53* or *CDKN2A* genes also represent risk factors for around 5% of PDAC patients [10,11]. Despite intensive research, the five-year overall survival (OS) rate for PDAC patients is around 7%, and one-year survival is achieved in less than 20% of cases [1,12]. The lack of effective therapies and chemotherapy resistance are crucial elements that contribute to this pejorative prognosis [13]. Early-stage PDAC are often asymptomatic which delays the diagnosis and treatment options. Importantly, efficacy and outcome of PDAC treatments are indeed determined by the disease stage at the time of diagnosis, which is done at an advanced stage most of the time. The only curative therapy available is surgical resection followed by adjuvant therapy [14], but unfortunately, 80% of PDAC patients have an advanced or metastatic disease that is ineligible to surgery [15]. Novel strategies for the identification of early-stage tumors and efficient targeted therapies have therefore gained valuable interest in recent years. Nevertheless, most clinical trials evaluating novel therapeutic approaches failed to demonstrate significant improvement of OS [16,17,18].

Among the relevant targets, accumulating evidences reveal mesothelin (MSLN) as a potential and promising biomarker of PDAC aggressiveness [19,20]. Importantly, MSLN expression is restricted to mesothelial cells and is dispensable in normal tissues. However, MSLN overexpression has been reported in a wide range of tumors, including 80 to 85% of PDAC [19,21,22]. The role of MSLN as a pro-tumorigenic factor and a therapeutic target in PDAC has thus gained a renewed interest. In this review, we first discuss the different functions of MSLN during PDAC progression, to finally emphasize on the MSLN-targeted agents that are currently under clinical development for diagnosis and PDAC treatment. 

## 2. Role of MSLN in PDAC Progression

### 2.1. Structure of MSLN and Physiological Functions

MSLN was identified by Ira Pastan and Mark Willingham thirty years ago [23,24]. The human *mesothelin* gene encodes a 71-kDa precursor protein, processed into a 31-kDa shed form (megakaryocyte-potentiating factor, MPF) and a 40-kDa glycosylphosphatidylinositol (GPI)-anchored membrane protein, MSLN. MSLN can also be processed by splicing or cleavage, to generate a truncated soluble form, the Serum Mesothelin Relative Peptide (SMRP) [25,26]. Three dimensional prediction programs suggested a super-helical structure with Armdillo-type repeats [27]. Although GPI-anchored proteins are generally involved in cell-cell adhesion or diverse signaling pathways, the biological function of MSLN still remains unknown. Indeed, *MSLN* gene inactivation did not reveal any developmental, anatomical nor histological abnormalities—no detectable phenotype is thus mirrored by the absence of *mesothelin* gene [28].

MSLN can bind to mucin MUC16 and this interaction mediates cellular adhesion [29,30]. MUC16 is a type-I transmembrane protein composed of a glycosylated extracellular N-terminal domain, tandem repeat domains and a C-terminal domain [31]. Interestingly, a recent study identified the potential role of MUC16-MSLN interaction in the regulation of liver fibrosis [32]. Overexpression of MUC16 has also been reported in several types of cancer including PDAC [33]. The interaction between MSLN on mesothelial cells, and MUC16 on ovarian cancer and PDAC cells was reported to favor peritoneal dissemination of tumors [30,33,34]. Muniyan et al. indeed reported that MUC16 knockdown not only slows-down in vitro proliferation and colony formation of tumorigenic PDAC cells (Capan-1 and colo-357), but also hampers in vivo tumorigenic potential—with reduced formation of pancreatic tumors and decreased metastatic dissemination [33].

### 2.2. Expression of MSLN in PDAC

**Figure 1:**
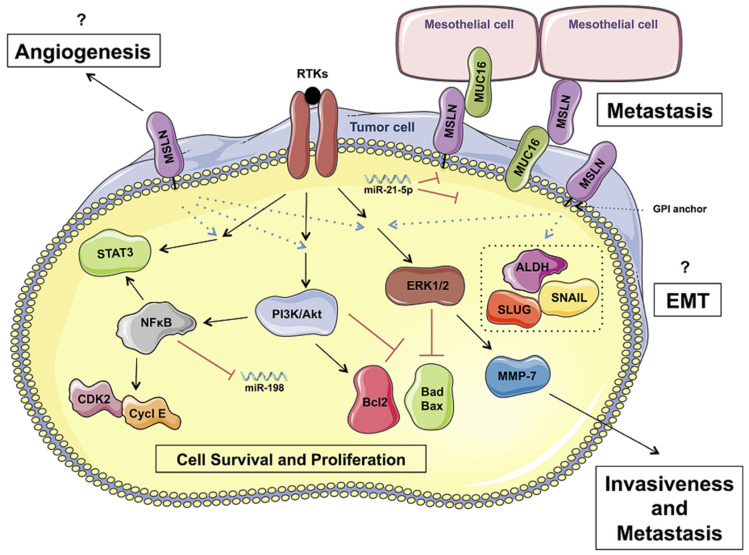
Putative roles of MSLN in PDAC progression. MSLN promotes tumor cell survival and proliferation through ERK and PI3K/Akt pathways; and invasiveness and metastasis processes by MMP-7 activity. Metastasis could also be facilitated by interaction with MUC16 expressing cells. However, the underlying mechanisms linking MSLN to EMT and angiogenesis, as well as the other putative partners of MSLN still remain to be elucidated in PDAC. The biological significances of miR-21-5p and miR-198 are also depicted in this schematic illustration.

MSLN expression has been evidenced by immunohistochemistry or microarray analyses in nearly 40% of solid tumors [19]. Initially, MSLN expression has been reported in 90% of mesothelioma and in 60 to 65% of ovarian cancers samples by Pastan’s team [23,35]. Similarly, MSLN was also reported in 25 to 67% of triple negative breast cancer and in 60 to 70% of lung cancers [36,37,38]. Importantly, besides these cancers, MSLN overexpression was also observed in 80 to 85% of PDAC-derived tumor samples [21,39,40,41,42]. However, MSLN is not expressed in para-cancer tissues samples [23,24,39,43]. PDAC progression is correlated with increased expression of MSLN especially in advanced-stage (stage IV) in comparison to early-stage (stage I) [43]. MSLN is a key player in sustaining intense cell proliferation, resistance to cell death, invasive and metastatic properties, and angiogenesis [19,22]. Nevertheless, all the partners implicated in these phenomena still remain to be discovered (Figure 1).

### 2.3. Cell Proliferation and Anti-Apoptosis

Li M et al. were the first to demonstrate that MSLN expression in PDAC was correlated with an increase of tumor volumes, thus suggesting an intense capacity of these tumorigenic cells to proliferate [44]. Through overexpression and downregulation of MSLN in Mia-Paca2 and BxPC-3 PDAC cells, Bharadwaj et al. further demonstrated a tight correlation between MSLN expression and the active form of STAT3 transcription factor [45]. Active STAT3, that is present in 30% of PDAC, enhanced the expressions of cyclin E and cyclin E/cyclin-dependent kinase 2 complex formation, thereby promoting increased G1-S transition [45]. MSLN also activates NFκB, which results in an increased IL-6 expression and cell proliferation, via an autocrine IL-6/sIL-6R trans-signaling [46]. The pro-proliferative effect of MSLN also involved ERK and PI3K/Akt pathways [47]. Indeed, silencing of MSLN in PDAC BxPC-3 cells decreased ERK1 (but not ERK2) and AKT activities that may probably explain its anti-proliferative effects considering the prominent role of ERK and AKT pathways during cell proliferation and metastasis processes.

MSLN signaling is also involved in the resistance to TNF-α-induced apoptosis through the activation of the Akt/PI3K/NFκB axis in PDAC [48]. In PDAC, MSLN decreases BAX and augments BCL-2, leading then to enhanced cell proliferation [49]. MSLN was also reported to be a key mediator of resistance to anoïkis—a form of programmed cell death that occurs in anchorage-dependent cells when they are detached from the surrounding extracellular matrix—especially through the activation of MEK1/2 pathway [50]. In breast cancer cells cultured in suspension, overexpression of MSLN (via ectopic expression) was reported to favor ERK1/2 in an active state and to decrease Bim levels—Bim being an important inducer of anoïkis. After treatment exposure with U0126—a MEK-1 inhibitor, the levels of phospho ERK1/2 and Bim were completely reversed, thereby confirming that the role sustained by MSLN during anoïkis resistance was intrinsically linked with the ERK1/2 pathway [50]. However, in PDAC, since no current data concerning these molecular mechanisms has so far been evidenced, it still remains to be elucidated.

### 2.4. Promoting Tumor Invasion and Metastasis

Overexpression of MSLN in 86% of metastatic tissues derived from ovarian cancers suggests its implication in the metastatic process [51]. As aforementioned, the interaction between MSLN and its unique known ligand MUC16 was suggested to play a key role during peritoneal dissemination, which is a step preceding hematogenic and lymphatic dissemination, in both ovarian cancers and PDAC [33,34]. In PDAC, MUC16 and MSLN expression is correlated, and this co-expression was shown to promote cell mobility and tumor invasion, especially through the activation of JNK and ERK pathways, and also through the matrix metalloproteinase (MMP)-7 activity [52,53]. MUC16-expressing PDAC treated with MSLN indeed displayed increased MMP-7 expression and invasive capacity, thus proposing that the MMP-7 induction and increased invasive potential could actually stem from the MSLN-MUC16 molecular interaction [53].

MSLN is also involved in the metastatic process through its role in epithelial-mesenchymal transition (EMT). EMT is defined by the acquisition of characteristics of invasive mesenchymal cells by epithelial cells [54]. EMT is involved in tumor invasiveness and metastasis and correlates with poor clinical outcomes in several types of solid tumors [55]. MSLN expression could favor EMT and stemness features [56]. Indeed, MSLN was shown to stimulate the expressions of ALDH, SNAIL, SLUG and TWIST, while decreasing expression of E-cadherin in lung carcinoma and mesothelioma cell lines. Inhibition of MSLN expression indeed revealed a reversion of this phenotype, with a significant decrease of metastatic nodules in rodents [56]. These last results might eventually suggest the same functionality of MSLN in PDAC, but it still remains to be elucidated.

Finally, MSLN-expressing tumors were also evidenced to be associated with a significant increase of microvascular density in newly formed pancreatic metastatic tissues—this would suggest a new potential role held by MSLN in promoting angiogenesis [57]. However, again, the underlying mechanisms leading to the angiogenesis process are still unknown and will therefore need to be illuminated.

### 2.5. Resistance to Chemotherapy

A tight association between resistance to chemotherapy and MSLN expression has been previously established [58,59]. Inhibition of MSLN in mesothelioma cell lines has been shown to restore cell sensitivity after exposure to cisplatin [58]. Clinical data also support a strong correlation between MSLN expression and resistance to chemotherapy in epithelial ovarian carcinoma patients [59]. Considering the overexpression of MSLN found in the vast majority of PDAC and the known and strong intrinsic resistance of PDAC patients to chemotherapy, such correlation would certainly deserve further investigations.

### 2.6. Genetic Regulation of MSLN Expression in Tumor Cells

A MSLN promoter called Canscript, located between −65 to −45 bp before the initiation transcription site of MSLN, has been identified in MSLN-expressing solid tumors [60]. This promoter is composed of two motifs: a conventional MCAT and a SP1-type motif [60]. However, the transcription factors that are involved in the regulation of MSLN gene expression still remain to be determined. Importantly, De Santi et al. recently demonstrated that MSLN expression was regulated by miR-21-5p [61]. miR-21-5p indeed acts as a tumor suppressor miRNA since it negatively regulates MSLN expression. The exact role of this microRNA is currently unclear, more particularly in PDAC and need further investigations. On the other side, Marin-Muller et al. demonstrated that MSLN represses miR-198 through a NFκB-dependent OCT-2 (homeobox transcription factors POU2F2) induction, leading then to increased tumorigenicity in PDAC. While reduced miR-198 expression predicted shorter survival, high miR-198 levels were associated with longer survival and better prognosis [62]. Given these last promising results, the roles of miRNAs during PDAC progression deserve to be further investigated.

## 3. Predictive Value of MSLN and Anti-MSLN Diagnosis-Dedicated Agents in PDAC

### 3.1. Predictive Value of MSLN Expression

The association between MSLN expression and clinical outcomes of ovarian, lung, breast cancers and PDAC patients has been extensively studied. MSLN and MUC16 co-expression, assessed by immunohistochemistry, was reported to be associated with increased tumor cell invasiveness and metastasis, and poor clinical outcomes of PDAC patients. Indeed, MUC16 high/MSLN high expression group not only displayed significantly reduced OS when compared to the other group, but also decreased disease-free survival, respectively 11.9 versus 22.8 months and 6.7 versus 10.9 months [52]. Of note, MUC16 and MSLN co-expression levels were found to be increased at the invasion front of PDAC-derived tissues in comparison to that of detected in the stroma—similarly, MUC16 high/MSLN high expression group was strongly associated with large tumors, invasion of other distant organs and lymphatic permeation [52].

### 3.2. SMRP Assay

The identification of soluble markers and the development of tests to reliably detect them represent major challenges in oncology. Such markers will be useful: (1) to enable early diagnosis, and (2) to evaluate the therapeutic efficacy of anti-cancer agents. Two soluble forms of MSLN have previously been identified: the MPF (32-kDa) and the 40-kDa form SMRP [24]. The diagnosis value of SMRP assays has been evaluated in ovarian cancers and mesothelioma. Therefore, high SMRP levels were evidenced in 75% of ovarian and mesothelioma cancer patients—a strong correlation between SMRP levels and cancer stages could then be demonstrated [63,64]. A quantitative assay for SMRP has been developed (Mesomark^®^) and validated for the detection and management of mesothelioma in clinical trials [65]. Unfortunately and despite the overexpression of membrane-associated MSLN in pancreatic cancers, elevated levels of SMRP has however not been reported in PDAC patients [66].

### 3.3. Imaging Probes for the Phenotyping of MSLN-Expressing Tumors

Some MSLN-targeting radiotracers serve as a companion marker of anti-MSLN therapies that are currently under clinical development. The different anti-MSLN therapies will be detailed in Section 4. In 1999, Hassan et al. were the first to evaluate the biodistribution of a 111-Indium (^111^In)-radiolabeled monoclonal antibody (mAb) in experimental models of ovarian cancer [67]. Interestingly, monoclonal anti-MSLN antibodies have also been evaluated for PDAC targeting in preclinical mouse models and in patients and specific binding to MSLN-expressing lesions was detected in mouse models of PDAC using ^64^Cu- and ^89^Zr- radiolabeled mAbs [68,69,70]. Clinical investigations could demonstrate high ability of ^111^In- and ^89^Zr- radiolabeled mAbs for phenotypic imaging of MSLN-expressing PDAC [71,72]. Nevertheless, the hepatic elimination of mAbs and their slow blood clearance constitute important limitations. Their use as imaging probes in clinical practices is indeed limited since good quality images can be acquired only a few days after the injection of mAbs. Finally, single-chain variant antibodies as well as single-domain antibodies demonstrating optimal pharmacokinetics have recently been validated for preclinical imaging of MSLN-expressing tumors, and will therefore deserve to be fully considered for further clinical development [43,73].

## 4. Anti-MSLN Targeting Drugs in Development

**Figure 2:**
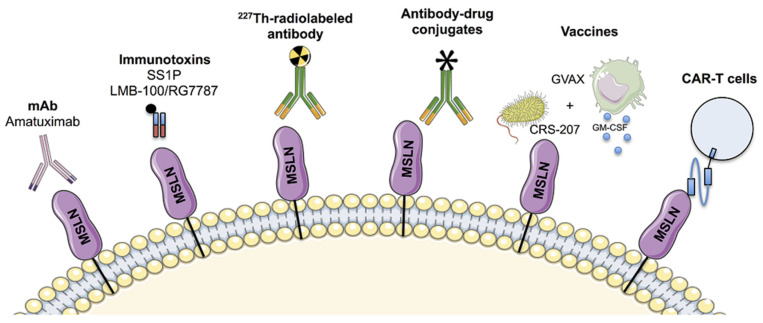
This schema depicts the different anti-MSLN targeting drugs that are currently under clinical development, including the monoclonal antibody (mAb) amatuximab, SS1P and LMB-100/RG7787 immunotoxins, ^227^Th-radiolabeled antibody, antibody-drug conjugates, vaccines and CAR-T-cells.

As discussed above, the limited expression of MSLN in normal tissues as well as its overexpression in a broad range of tumors makes MSLN an attractive and promising target for therapy. Hence, antibody-based drugs, vaccines or also CAR-T cells have been developed to target MSLN-expressing tumors (Figure 2). Also, most of these strategies have been assessed in preclinical and clinical trials. It is important to note here that anti-MSLN antibodies have frequently been identified to be present in blood samples of patients with mesothelioma and ovarian cancers, thereby advocating MSLN as a valuable immunogenic protein [74].

### 4.1. Antibodies-Based Approach

#### 4.1.1. Immunotoxins SS1P and LMB-100/RG7787

SS1 is an anti-MSLN Fragment antigen binding (Fab). The fusion of SS1 to a *Pseudomonas* exotoxin (PE38) resulted in the recombinant immunotoxin (RIT) called SS1P. SS1P is internalized into MSLN-expressing cells and induces apoptosis by inactivation of the elongation factor 2 [75]. The anti-tumor effect of SS1P alone or in combination with radiotherapy or chemotherapeutic agents has been validated by several preclinical studies [76,77]. Based on these encouraging results, SS1P has been evaluated in several clinical trials enrolling patients with advanced cancers. A phase I trial including 33 patients (mesotheliomas, ovarian cancer and PDAC) demonstrated the absence of pericardial toxicity, thereby suggesting that the SS1P-induced damages are limited, with a reduced risk for mesothelial cells [78]. Among the 33 patients, 4 displayed minor response, 19 had stable disease (SD) and 10 had progressive disease (PD). Nevertheless, the efficacy of SS1P was limited due to the formation of anti-SS1P neutralizing antibodies in 88% of patients. In an attempt to increase SS1P efficacy and also to limit the development of anti-SS1P antibodies, a phase I trial on SS1P-treated mesothelioma patients in combination with pemetrexed and cisplatin immunosuppressive drugs was conducted. Using the Response Evaluation Criteria in Solid Tumors (RECIST), partial response (PR) was obtained in 10 out of 13 patients and was associated with limited levels of anti-SS1P antibodies. [79]. In chemotherapy-refractory mesothelioma, 3 out of 10 patients had major tumor regression following SS1P and pentostatin administration [80]. Therefore, based on these promising results, a phase I/II trial in mesothelioma and PDAC patients has recently been conducted and results are currently awaited (NCT01362790).

In order to limit the formation of neutralizing antibodies, a second strategy relying on the modification of SS1P sequences has also been evaluated. LMB-100/RG7787, a re-engineered version of SS1P combining SS1 Fab with the truncated and deimmunized PE24 moiety, was shown to restrict tumorigenicity both in vitro and in vivo using preclinical models of PDAC [81]. Similarly, its combination with taxanes, actinomycin D and panobinostat were also associated with increased anti-tumor activities in both mesothelioma and PDAC preclinical models—LMB-100/RG7787 is then currently under clinical trial for these types of cancer (NCT02810418) [82,83,84]. LMB-100/RGG7787 combined with tofacitinib, is also under clinical investigation for the treatment of MSLN-expressing tumors, including PDAC (NCT04034238).

#### 4.1.2. Monoclonal Antibody: Amatuximab

Amatuximab (or MORAb-009) is a chimeric high-affinity mAb targeting MSLN inhibiting MUC16/MSLN interaction. Amatuximab eradicates tumor cell through antibody-dependent cellular cytotoxicity [85], and exerts a strong anti-tumoral effect when combined with gemcitabine in a peritoneal metastasis mouse model of PDAC [86]. Moreover, a phase I clinical trial in patients with MSLN-expressing tumors, including PDAC, revealed absence of any severe adverse events [87]. However, among 20 out of 24 available patients, none of them exhibited complete or PR, therefore limiting the use of amatuximab alone.

Based on this favorable safety profile, a phase II clinical trial investigating the effect of amatuximab in combination with pemetrexed and cisplatin has been conducted in 89 mesothelioma patients. A slight increase of the OS was then observed in comparison to controls [88]. Moreover, in mesothelioma patients, a higher exposure of amatuximab in combination with chemotherapy regimen is associated with significant longer OS [89].

#### 4.1.3. Antibody-Drug Conjugates

Several antibody-drug conjugates (ADC) targeting MSLN have been developed. ADC relies on the specificity of an antibody with the cytotoxic potential of a chemotherapeutic agent. For example, anetumab ravtansine or BAY94-9343 is an anti-MSLN antibody coupled to the maytansinoid tubulin inhibitor DM4 via a disulfide-containing linker. Hence, anetumab ravtansine was reported to display promising anti-tumor activity in preclinical models of PDAC by inducing bystander effects [51]. Moreover, in a recent phase Ib, anetumab ravtansine exhibited favorable safety profile and encouraging anti-tumor activity in patients with MSLN-expressing tumors, including PDAC [90].

Several phase I clinical studies investigating the safety of anetumab ravtansine in combination with pembrolizumab (anti-programmed cell death (PD-1)) or atezolizumab, (anti-programmed cell death ligand 1 (PDL-1)) antibodies, are ongoing (NCT03455556, NCT03126630). In advanced PDAC, a phase II clinical trial with anetumab ravtansine is currently ongoing in the United States (NCT03023722).

DMOT4039A is a humanized anti-MSLN mAb fused to monomethyl auristatin (MMAE), an anti-mitotic agent that blocks the polymerization of tubulin. A single-dose of DMOT4039A prevented tumor growth in a dose-dependent manner in murine models of ovarian cancer, PDAC and mesothelioma, as well as in mice bearing patient-derived tumor xenografts [91]. Also, the safety profile of this agent was proved in a phase I clinical trial carried out on 71 PDAC and ovarian cancer patients [92]. A total of 6 patients (4 of ovarian cancer and 2 of PDAC) had a confirmed PR.

BMS-986148 is a mAb conjugated to the alkylating agent duocarmycin, leading to the death of MSLN-expressing cells after internalization, and it is currently undergoing a phase I clinical trial [93]. The evaluation of the safety, tolerability, pharmacokinetics, pharmacodynamics as well as anti-tumor activity are about to be evaluated in MSLN-expressing solid tumors, including PDAC (NCT02341625). Initial observations for BMS-986148 in combination with nivolumab from a phase I/IIa clinical trial have pinpointed the relevance of such therapies in reducing the tumorigenic potential of MSLN-positive tumors [94].

#### 4.1.4. ^227^Th-Radiolabeled Antibody

Targeted alpha therapy is a type of targeted radionucleide therapy that employs high-energy emission of short-range alpha particles to favor and induce cell death. The short-range emission of these particles confines its cytotoxic effect in tumor cells that are specifically targeted, thereby limiting damages to the surrounding healthy tissues [95]. Targeted 227-Thorium (^227^Th) conjugates thus represent a new and promising class of therapeutics for targeted alpha therapy. A ^227^Th-radiolabeled mAb directed against MSLN, the BAY2287411, has recently been assessed in combination with DNA damage response inhibitors using experimental ovarian cancers—this combination revealed a synergistic anti-tumor effect [96]. Moreover, a Phase I study using BAY2287411 conducted in advanced mesothelioma and ovarian cancer patients, and in patients with metastatic or locally advanced PDAC is currently ongoing (NCT03507452).

### 4.2. Cancer Vaccines

Cancer vaccines are designed to induce tumor-specific immune response. Elizabeth Jaffee’s team was the first to identify MSLN as immunogenic protein, using GVAX (GM-CSF gene-transfected tumor cell vaccine), which originates from irradiated PDAC cells genetically engineered to secrete the granulocyte-macrophage colony-stimulating factor (GM-CSF). GVAX induced a T-cell response against several antigens including MSLN. Enhanced MSLN-specific CD8 T-cell responses were detected in GVAX treated PDAC patients and associated with longer OS [18]. Subsequent clinical studies demonstrated that MSLN-specific CD8+ T cell response was associated with improved disease free survival and OS in patients with metastatic PDAC [97,98]. On the other side, CRS-207, a vaccine based on the *Listeria monocytogenes* bacterial strain ANZ-100 engineered to express MSLN, has been developed. CRS-207 was evaluated in a dose-escalation study in a total of 17 patients including 7 PDAC and revealed that despite its safety profile and ability to induce immune activation, none of the patients displayed a PR [99]. Interestingly, CRS-207 has also been evaluated in combination with GVAX and cyclophosphamide in 90 previously treated PDAC patients. Patients treated with this combined treatment displayed significant longer OS in comparison with patients that have received cyclophosphamide and GVAX, respectively 6.1 versus 3.9 months [18]. Unfortunately, a phase IIb conducted on 303 metastatic pre-treated PDAC patients that were administrated with GVAX + CRS-207, CRS-207 or chemotherapy alone failed to demonstrate any improvement of OS [100].

Other MSLN-targeted vaccines are actually under preclinical development such as the Meso-VAX. Meso-VAX enhanced MSLN-specific T cell populations and anti-MSLN antibodies production when administrated in combination with the adeno-associated virus expressing IL-12 [101]. Recently, a novel HLA-A24-restricted epitope derived from MSLN was shown to induce peptide-specific cytotoxic T lymphocytes—these cytotoxic T lymphocytes indeed demonstrated a specific toxicity against MSLN expressing PDAC [102].

Although cancer vaccines induce an effective anti-tumor T cell response, their use alone is insufficient to inhibit tumor growth. The up-regulation of PD-L1 has been reported in PDAC and the PD1/PD-L1 signaling pathway was shown to hamper the efficiency of anti-tumor immune response [103]. Importantly, PD-L1 was reported to be upregulated in PDAC patients treated with therapeutic vaccines [104]. Hence, a randomized phase II study that aimed at evaluating the safety, efficacy and immune response to GVAX in metastatic PDAC with cyclophosphamide and CRS-207, with or without the anti PD-1 nivolumab, has recently been published (NCT02243371). However, this study failed to demonstrate any improvement of OS [105].

### 4.3. CAR-T Cells

Among immunotherapies, chimeric antigen receptor T cells (CAR-T) therapy has been described as being one of the most promising novel approaches in order to treat cancer. CAR-T cells are engineered T cells that produce an artificial T-receptor targeting a specific protein. The CARs are composed of an extracellular antigen binding domain, a transmembrane domain and an intracellular domain transmitting T cell activation signals. The first-generation of CARs consisted of only one intracellular signaling domain, a CD3z chain [106]. Despite their ability to activate T-cells, they did not produce a long-term proliferative activity. The second-generation of CARs was composed of a co-stimulatory molecule (CD28 or 4-1BB), thereby maintaining a long-term proliferative ability [106]. Later on, several teams demonstrated that the third generation of CARs containing two costimulatory domains was significantly superior to the previous generations in terms of cytotoxicity, persistence and anti-tumor activity. Finally, the fourth generation of CARs favors cytokine secretion (including IL-12, IL-15)), thereby strongly influencing the immune component of the tumor microenvironment [107]. Two years ago, the first CAR-T therapy, targeting CD19 (Kymriah^TM^, tisagenlecleucel) has been approved by the Food and Drug administration and the European Medicine Agencies for the management of patients with acute lymphoblastic leukemia [108]. However, treatment of solid tumors with CAR-T cells is facing several obstacles including the hostile tumor microenvironment or even on-target/off-tumor toxicities. Some anti-MSLN CAR-T cells have been developed during the last 5 years. These engineered T cells infiltrate PDAC tumors and mediate potent anti-tumor activity [109].

Preclinical studies performed on mouse models of metastatic PDAC demonstrated that MSLN-directed CAR-T cells induced tumor cytotoxicity and could eradicate lung metastases [110,111]. Regional intrapleural injection of MSLN-directed CAR-T cells resulted in an efficient anti-tumor activity in association with their proliferation and persistence after 200 days using an orthotopic mouse model of mesothelioma [112]. Similar results were recently reported after injection of MSLN-directed CAR-T cells in the peri-tumoral zone using preclinical model of gastric cancers [113].

Currently, the majority of clinical trials that target MSLN-expressing tumors relies on CAR-T cell therapy. Several phase I/II clinical trials are currently ongoing in PDAC patients, aimed at determining the safety, the anti-tumoral effects and the maximum tolerated dose. In a phase I clinical study, 6 patients with metastatic PDAC were intravenously injected with anti-MSLN CAR-T cells twice per week during three weeks, and no side effects or dose-limiting toxicities were reported [114]. Moreover, anti-MSLN directed CAR-T cells in combination with cyclophosphamide is currently under phase I clinical study in 18 PDAC patients (NCT03323944). As preclinical studies demonstrated the higher persistence of regionally injected CAR-T cells, several clinical studies are presently exploiting the possibility to infuse CAR-T cells in PDAC through vascular intervention in order (1) to potentiate the drug concentration of the tumor site, and (2) to reduce the potential off-target side effects (NCT02706782, NCT03267173). Finally, the safety and efficacy of CTLA-4 and PD-1 expressing MSLN-CAR-T cells are currently in clinical evaluation in MSLN-expressing PDAC patients (NCT03615313, NCT03182803) (Table 1).

While CAR-T cell approaches appear to be an appealing strategy, serious side effects such as cytokine release syndrome or anaphylactic shock may however decrease their efficiency. To avoid these complications, a pro-apoptotic suicide gene (iCaspase9) has recently been included—such strategy is actually considered as a possible approach to increase anti-MSLN CAR-T cell tolerance [115]. Finally, a MSLN-targeted CAR-T cells therapy trial is ongoing using iCaspase9 (NCT03747965).

## 5. Conclusions

The limited physiological expression of MSLN and its overexpression in a broad range of tumors make it an attractive target for therapy in several cancers including PDAC. Despite high membrane-associated MSLN expression in PDAC, soluble MSLN assay failed in validating their diagnostic potential for PDAC. Nuclear imaging agents currently in development will serve as companion markers for MSLN-targeted therapies. While MSLN-targeted therapies displayed acceptable safety profile, their anti-tumoral efficacy is often limited. However, MSLN, in spite of an incompletely resolved mode of action, remains a very attractive target and several clinical trials are currently ongoing and the medical community awaits their final results with much interest. To take this reasoning further, it is increasingly evident that the benefit of single-agent therapies becomes limited and future directions will necessarily involve combined therapies especially with immune checkpoint inhibitors, immune-stimulation as well as radiotherapy or radio-sensitizing agents.

## Figures and Tables

**Table 1:** Clinical trials for MSLN-targeted therapies in PDAC.

Agent	Title	Status	Phase	NCT/References
**SS1P**	Phase I study of SS1P, a recombinant anti-mesothelin immunotoxin given as a bolus I.V. infusion to patients with mesothelin-expressing mesothelioma, ovarian, and pancreatic cancers.	Completed (Results available)	I	[78]
**LMB-100/RG7787**	Mesothelin-targeted immunotoxin LMB-100 alone or in combination with Nab-Paclitaxel in people with previously treated metastatic and/or locally advanced PDAC and mesothelin-expressing solid tumors.	Active	I/II	NCT02810418
	Mesothelin-targeted immunotoxin LMB-100 in combination with tofacitinib in persons with previously treated metastatic PDAC and other mesothelin-expressing solid tumors.	Recruiting	I	NCT04034238
**Amatuximab**	Phase I clinical trial of the chimeric anti-mesothelin monoclonal antibody MORAb-009 in patients with mesothelin-expressing cancers.	Completed (Results available)	I	[87]
**Anetumab ravtansine**	First-in-Human, Multicenter, Phase I Dose-Escalation and Expansion Study of Anti-Mesothelin Antibody-Drug Conjugate Anetumab Ravtansine in Advanced or Metastatic Solid Tumors.	Completed (Results available)	Ib	[90]
	Phase II anetumab ravtansine in pre-treated mesothelin-expressing pancreatic cancer	Completed	II	NCT03023722
**DMOT4039A**	Phase I study of DMOT4039A, an antibody-drug conjugate targeting mesothelin, in patients with unresectable pancreatic or platinum-resistant ovarian cancer.	Completed (Results available)	I	[92]
**BMS-986148**	A study of BMS-986148 in patients with selected advanced solid tumors.	Completed	I/II	NCT02341625
**BAY2287411**	First in human study of BAY2287411 injection, a Thorium-227 labeled antibody-chelator conjugate, in patients with tumors known to express mesothelin.	Recruiting	I	NCT03507452
**CRS-207**	A live-attenuated Listeria vaccine (ANZ-100) and a live-attenuated Listeria vaccine expressing mesothelin (CRS-207) for advanced cancers: phase I studies of safety and immune induction.	Completed (Results available)	I	[99]
	Results from a Phase IIb, Randomized, Multicenter Study of GVAX Pancreas and CRS-207 Compared with Chemotherapy in Adults with Previously Treated Metastatic Pancreatic Adenocarcinoma (ECLIPSE Study).	Completed (Results available)	IIb	[100]
	Evaluation of Cyclophosphamide/GVAX Pancreas Followed by Listeria-mesothelin (CRS-207) With or Without Nivolumab in Patients with Pancreatic Cancer.	Completed (Results available)	II	[105]
**CAR-T cells**	Activity of mesothelin-specific chimeric antigen receptor T cells against pancreatic carcinoma metastases in a phase 1 trial.	Completed (Results available)	I	[114]
	CAR T Cell immunotherapy for pancreatic cancer.	Active	I	NCT03323944
	A study of mesothelin redirected autologous T cells for advanced pancreatic carcinoma.	Unknown	I	NCT02706782
	Evaluate the safety and efficacy of CAR-T in the treatment of pancreatic cancer.	Unknown	I	NCT03267173
	PD-1 antibody expressing mesoCAR-T cells for mesothelin positive advanced solid tumor.	Recruiting	I/II	NCT03615313
	CTLA-4 and PD-1 antibodies expressing mesothelin-CAR-T cells for mesothelin positive advanced solid tumor.	Unknown	I/II	NCT03182803
	Study of PD-1 gene-knocked out mesothelin-directed CAR-T cells with the conditioning of PC in mesothelin positive multiple solid tumors.	Recruiting	I	NCT03747965

## Abbreviations

ADC	Antibody drug-conjugates
CAR-T	Chimeric antigen receptor T cells
Fab	Fragment antigen binding
GM-CSF	Granulocyte-macrophage colony-stimulating factor
GPI	Glycosylphosphatidylinositol
GVAX	GM-CSF gene-transfected tumor cell vaccine
mAb	Monoclonal antibody
MAV	Metabolic Active Volume
MPF	Megakaryocyte-potentiating factor
MSLN	Mesothelin
MUC16	Mucin 16
OCT-2	Homeobox transcription factors POU2F2
OS	Overall survival
PD-1	programm death 1
PD-L1	programm death ligand 1
PD	Progressive disease
PDAC	Pancreatic ductal adenocarcinoma
PR	Partial response
RECIST	Response Evaluation Criteria in Solid Tumors
RTK	Receptor Tyrosine Kinase
SD	Stable disease
SMRP	Serum mesothelin-related peptide
Tat	Targeted alpha therapy
